# Increased monocyte level is a risk factor for radiological progression in patients with early fibrotic interstitial lung abnormality

**DOI:** 10.1183/23120541.00226-2022

**Published:** 2022-07-04

**Authors:** Andrew Achaiah, Paul Lyon, Emily Fraser, Peter Saunders, Rachel Hoyles, Rachel Benamore, Ling-Pei Ho

**Affiliations:** 1 MRC Human Immunology Unit, Weatherall Institute of Molecular Medicine, University of Oxford, Oxford, UK; 2 Oxford Interstitial Lung Disease Service, Oxford University Hospitals NHS Foundation Trust, Oxford, UK; 3 Oxford Radiology Unit, Oxford University Hospitals NHS Foundation Trust, Oxford, UK

## Abstract

**Background:**

Interstitial lung abnormalities (ILA) are specific spatial patterns on computed tomography (CT) scan potentially compatible with early interstitial lung disease. A proportion will progress; management involves risk stratification and surveillance. Elevated blood monocyte levels have been shown to associate with progression of idiopathic pulmonary fibrosis. The aims of the present study were: 1) to estimate the proportion of “early fibrotic” (EF)-ILAs (reticular±ground-glass opacities, excluding traction bronchiectasis and honeycombing) on CT scans of patients attending all-indications thoracic CTs, and proportion demonstrating radiological progression; and 2) to explore association between peripheral blood leukocyte levels and ILA progression.

**Methods:**

We analysed all thoracic CT reports in individuals aged 45–75 years performed between January 2015 and December 2020 in one large teaching hospital (Oxford, UK) to identify patient CT reports consistent with EF-ILA. CT-contemporaneous blood leukocyte counts were examined to explore contribution to progression and all-cause mortality, using multivariate Cox regression.

**Results:**

40 711 patients underwent thoracic CT imaging during this period. 1259 (3.1%) demonstrated the EF-ILA pattern (mean±sd age 65.4±7.32 years; 735 (47.8%) male). EF-ILA was significantly associated with all-cause mortality (hazard ratio 1.87, 95% CI 1.25–2.78; p=0.002). 362 cases underwent at least one follow-on CT. Radiological progression was observed in 157 (43.4%) cases: increase in reticulation n=51, new traction bronchiectasis n=84, honeycombing n=22. Monocyte count, neutrophil count, monocyte:lymphocyte ratio, neutrophil:lymphocyte ratio and “systemic inflammatory response index” were significantly associated with radiological progression.

**Conclusion:**

3.1% of subjects requiring thoracic CT during a 6-year period demonstrated EF-ILA. Monocyte levels and blood leukocyte-derived indexes were associated with radiological progression and could indicate which patients may require closer follow-up.

## Abstract 2

**Monocyte levels are associated with radiological progression of early fibrotic ILA to established interstitial lung disease and could indicate which patients might require closer follow-up** https://bit.ly/3LlS2ff

## Introduction

Interstitial lung abnormalities (ILA) refer to specific spatial patterns on computed tomography (CT) scan that are potentially compatible with interstitial lung disease (ILD) in individuals where ILD was not previously suspected [1]. As a proportion of ILAs are detected coincidentally in asymptomatic individuals, it is difficult to determine its prevalence. However, large cohort studies have reported prevalence of 2–10% [2–5] and a higher risk of mortality [2, 6]. ILAs have been shown to be associated with symptoms including breathlessness, reductions in lung function [2] and exercise capacity [7] and genetic abnormalities common to familial interstitial pneumonia and idiopathic pulmonary fibrosis (IPF) [8, 9]. A proportion of ILAs are known to progress to IPF, yet ILA prevalence exceeds that of IPF by a considerable margin. Therefore, identifying cases at risk of progression is an important clinical priority [10].

Future implementation of lung cancer screening and greater use of CT imaging for other diagnostic purposes are likely to increase detection of ILA and pose resource implications for ILD services [11]. A recent position paper on ILA from the Fleischner Society [1] discussed risk stratification, schema for follow-up evaluation and the importance of subcategorising for the subpleural fibrotic ILA, which has greater mortality risk.

Earlier detection of fibrotic ILAs could lead to a shorter lag time to ILD diagnosis, potentially allowing earlier treatment intervention and improved patient outcome [12]. Perhaps more pressing is a need for an easily accessible test to stratify patients according to those who are more likely to progress and therefore require follow-up.

Measurement of peripheral blood leukocytes for prognosis purposes in IPF has gained interest in recent years [13–15]. Furthermore, indexes derived separately from peripheral blood leukocytes have demonstrated correlation with adverse clinical outcomes in ILD [16].

In this study, we examined the prevalence of ILA with early fibrotic features in a population over a 6-year period. Focusing on radiographic appearances, we identified a group of ILAs, which we termed “early fibrotic ILA” (EF-ILA), defined as those with reticular±ground-glass presence and excluding traction bronchiectasis and honeycombing; and questioned whether there is an association between leukocyte profile and outcome, focusing on mortality and radiological progression (in extent, and with emergence of traction bronchiectasis and honeycombing) [17].

## Methods

Using the UK National Health Service (NHS)-based Clinical Record Interactive Search database of the Oxford University Hospitals NHS trust (estimated catchment population of 800 000; Oxfordshire, UK), we examined available CT reports for all thorax-protocolised CT scans performed between January 2015 and December 2020. We performed a starting keyword search using criteria selective for parenchymal abnormalities with an early fibrotic pattern: [“reticulation” or “interstitial”] AND [“sub-pleural” or “basal” or “lower zone” or “possible UIP”] AND [age range: 45–75]. Further details are provided in the supplementary methods.

We then screened the preliminary search for additional radiographic features: ground-glass opacities (GGO), traction bronchiectasis and honeycombing, in acknowledgment that multiple parenchymal features can co-exist on CT [1, 18]. Cases with or without GGO, but without traction bronchiectasis and honeycombing were termed EF-ILA. Those with traction bronchiectasis and honeycombing were classified as CTs showing traction bronchiectasis and/or honeycombing. We considered that these were representative of established fibrosis and/or usual interstitial pneumonia (UIP) pattern fibrosis, and not ILAs. In a proportion of cases identified from the preliminary keyword search, we later found on screening that CT reports were detailing negative/absence of specific radiological patterns; these were defined as a “nil-ILA” reference cohort. These cases were separated from the EF-ILA cohort.

Demographic data were collated, including age, gender and comorbidity profiles.

Over this period, patients who had more than one CT scan were identified, and the reports of the earliest and latest CT scans analysed for radiographic progression. Radiographic progression was recorded as a binary event. It was defined as increase in either extent of identified early fibrotic features (reticulation and/or GGO), new emergence of traction bronchiectasis and/or new emergence of honeycombing. In cases not demonstrating radiographic progression, this was defined as unchanged pre-existing parenchymal features and absence of new features. Time interval between first and latest CT was calculated. Mortality was recorded and time from first CT was calculated. For those who survived, a censoring date of 1 April 2021 was used.

Blood leukocyte counts (monocytes, neutrophils and lymphocytes) closest to the CT scan were recorded and monocyte:lymphocyte ratio (MLR), neutrophil:lymphocyte ratio (NLR) and systemic inflammatory response (SIRI: (monocytes×neutrophils) ÷ lymphocytes) indexes were calculated [19].

### Statistical analysis

Where relevant, tests for normality of data were performed using a D'Agostino–Pearson test, and following this the difference between groups was analysed using unpaired t-tests or the Mann–Whitney test for parametric and nonparametric analysis, respectively. Contingency tests (Fisher's exact test of significance) were used to assess categorical data.

Cox proportional hazard models were used to determine hazard ratios (HRs) to progression and all-cause mortality (separate models). In both models, age, gender, and monocyte, neutrophil and lymphocyte levels obtained at a time point closest to the CT scan were included. Coefficient of variation values for each case were calculated from available counts for monocytes, neutrophils and lymphocytes (and derived indexes) in the 1 year up to first CT to account for within-group variance in these measures. ILA categories were included in regression models and where stated hazard ratios represent either absolute floating risk or expressed relative to the nil-ILA category (reference category). Hazard ratios generated from continuous covariates represented the change in the risk of outcome if the covariate in question changes by one unit. Statistical significance was performed using the likelihood ratio test.

Reported statistical confidence intervals are at 95%. Two-tailed p-values <0.05 determined statistical significance. All analyses were performed using GraphPad Prism (version 9) or SPSS (version 26; IBM, Armonk, NY, USA).

### Ethical approval

The study was part of a study to examine the factors associated with disease progression in IPF (ethical approval 14/SC/1060 from the Health Research Authority and South-Central National Research Ethics Service).

## Results

### CT-based patient categorisation and demographics

170 197 CT scans were performed, which included any thoracic CT protocol between January 2015 and December 2020 in 40 711 patients. 3987 CT scans (performed in 2735 patients) satisfied the starting search criteria of [“reticulation” or “interstitial”] AND [“sub-pleural” or “basal” or “lower zone” or “possible UIP”] AND [age range: 45–75]. 355 cases did not demonstrate ILD or ILA and were used as a non-ILA reference cohort. 762 cases also demonstrated additional traction bronchiectasis and or honeycombing present on their first CT. 490 cases demonstrated traction bronchiectasis only; 270 demonstrated honeycombing±traction bronchiectasis.

1259 (3.1%) out of 40 711 cases demonstrated reticulation±GGO, without traction bronchiectasis or honeycombing. 430 also had emphysema and 88 also had non-emphysematous cysts. Therefore, 3.1% of subjects (1259 of the starting cohort of 40 711) requiring thoracic CT between 2015 and 2020 demonstrated EF-ILA. Mean±sd age of the EF- ILA group was 65.4±7.32 years; 735 (47.8%) were male.

**FIGURE 1:**
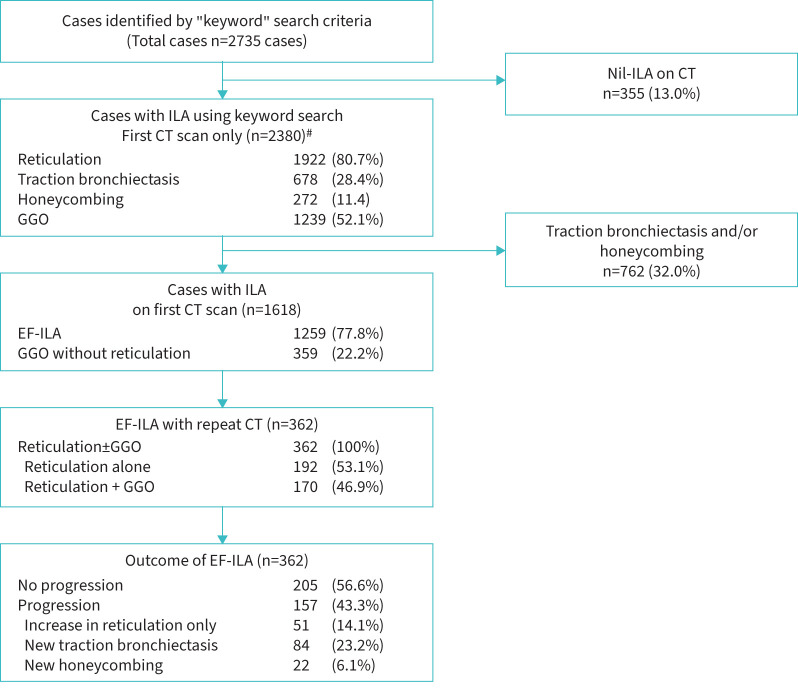
Flow diagram of interstitial lung abnormalities (ILA) features and radiological progression of cases with early fibrotic ILA (EF-ILA). Where more than one computed tomography (CT) scan was performed during the observation period, the first CT scan was used as the CT scan for the patient. GGO: ground-glass opacities. ^#^: a proportion of first CT scans demonstrated two or more ILA features simultaneously.

Demographic profiles are listed in table 1 and a flow chart of ILA features and progression is shown in figure 1. Comorbidity profiles were identified by cross-referencing electronic health records. Comorbidities are representative of the time the search was conducted and not at time of first CT.

**TABLE 1:** Demographic and blood leukocyte profiles of patients with no interstitial lung abnormalities (ILA) and early fibrotic (EF)-ILA

	**No ILA**	**EF-ILA**
**Demographics**		
Female	152 (42.8)	657 (52.2)
Male	203 (57.2)	602 (47.8)
Age at first CT (years)	63.4±8.1	65.39±7.32
**Comorbidity**		
COPD/emphysema	52 (14.6)	306 (19.9)
Pneumonia	70 (19.7)	344 (22.4)
Lung cancer	30 (8.5)	183 (11.9)
Pulmonary hypertension	13 (3.7)	68 (4.4)
T2DM	57 (16.1)	259 (16.8)
Hypertension	172 (48.5)	664 (43.2)
IHD	66 (18.6)	289 (18.8)
Cardiomyopathy	115 (32.4)	412 (26.8)
**Blood leukocyte measurements**		
Time from CT to nearest blood test (months)		
Mean±sd	0.87±5.92	0.78±6.29
Median (interquartile range)	0.11 (−0.23–1.24)	0.10 (−0.39–1.11)
Monocyte (×10^9^ cells·L^−1^)	0.65±0.29	0.67±0.31
Neutrophil (×10^9^ cells·L^−1^)	5.46±3.30	5.24±3.00
Lymphocyte (×10^9^ cells·L^−1^)	1.76±0.92	1.94±3.60
MLR	0.46±0.39	0.45±0.36
NLR	4.46±5.79	3.92±5.22
SIRI	3.21±5.74	2.75±4.33
**Seen in ILD clinic**	38 (10.7)	343 (27.2)
Length of follow-up (months)	21.46±21.7	23.4±21.8
Time from first CT to ILD clinic visit (months)	16.75±16.8	37.4±160.9

Data are presented as n (%) or mean±sd, unless otherwise stated. CT: computed tomography; T2DM: type 2 diabetes mellitus; IHD: ischaemic heart disease; MLR: monocyte:lymphocyte ratio; NLR: neutrophil:lymphocyte ratio; SIRI: systemic inflammatory response ((monocytes×neutrophils) ÷ lymphocytes); ILD: interstitial lung disease.

3217 CT scans (80.7% of scans with available information) were reported by a thoracic radiologist.

343 cases with EF-ILA on first CT were seen in the ILD clinic. Mean time from CT to clinic attendance was 3.1 years.

### Radiological progression on follow-on CT

Of the 1259 cases with EF-ILA, 362 patients underwent at least one follow-on CT scan, allowing examination of radiological change. Median (interquartile range (IQR)) time interval between CTs was 0.83 (0.32–1.95) years. Progression in type or extent of ILA was observed in 157 (43.4%) cases. Of these, increase in reticulation was observed in 51 (14.1%) out of 362 cases. Progression with emergence of traction bronchiectasis (excluding honeycombing) was observed in 84 (23.2%) cases, and honeycombing (with or without traction bronchiectasis) in 22 (6.1%) cases. 205 (56.6%) did not progress during this time (up to 5 years).

Median time interval between CT scans in cases demonstrating progression was 1.24 years (IQR 0.53–2.36 years, maximum 5.20 years) *versus* 0.59 years (IQR 0.27–1.19 years, maximum 5.05 years) in cases demonstrating no progression (p<0.001).

Unsurprisingly, multivariate Cox regression analysis showed that radiographic progression of EF-ILA was associated with mortality (HR 1.92, 95% CI 1.51–3.21; p=0.013).

### Imaging features and ILA mortality

Death was reported in 448 (16.40%) cases in the 6 years of analysis. Mean±sd time from first CT to death was 19.8±16.5 months *versus* 35.6±20.6 months in those who survived (p<0.0001). In cases with EF-ILA, death was reported in 183 cases. Mean±sd time from first CT to death was 19.0±16.6 months *versus* 32.7±20.5 months in those who survived.

Association between specific ILA features noted on first CT and mortality was explored using multivariate Cox regression (table 2). As expected, traction bronchiectasis (HR 2.09, 95% CI 1.36–3.20; p=0.0001) and honeycombing (HR 3.65, 95% CI 2.38–5.60; p<0.0001) were significantly associated with mortality relative to the nil-ILA reference category. Cases with EF-ILA also demonstrated significant mortality risk (HR 1.87, 95% CI 1.25–2.78; p=0.002). This risk was slightly higher in cases of EF-ILA that also demonstrated GGO (HR 2.03, 95% CI 1.29–3.19; p=0.002) in comparison to cases of EF-ILA without GGO (HR 1.80, 95% CI 1.19–2.72; p=0.005). Mortality risk of EF-ILA was preserved when adjusting for the respiratory comorbidities: lung cancer, pneumonia and COPD/emphysema. Lung cancer and pneumonia were also significantly associated with mortality in the EF-ILA group (supplementary table S1).

**TABLE 2:** Multivariate Cox regression examining association of interstitial lung abnormalities (ILA) features on first computed tomography (CT) scan with mortality

	**Patients**	**Death**	**HR (95% CI)** ^#^	**p-value**
**Age**			1.02 (1.01–1.04)	0.010*
**Gender (male)**	1486 (54.3)	268 (18.0)	1.11 (0.91–1.36)	0.295
**Nil-ILA (reference)**	355 (12.9)	43 (12.1)		
**EF-ILA**	1259 (46.0)	183 (14.5)	1.87 (1.25–2.78)	0.002*
**Traction bronchiectasis without honeycombing**	490 (17.9)	86 (17.6)	2.09 (1.36–3.20)	0.0001*
**Honeycombing±traction bronchiectasis**	272 (9.9)	87 (32.0)	3.65 (2.38–5.60)	<0.0001*

Data are presented as n (%), unless otherwise stated. Findings were similar when adjusted for respiratory comorbidities (supplementary table S1). HR: hazard ratio. EF-ILA: early fibrotic ILA. ^#^: HR for ILA categories representative of risk relative to nil-ILA reference category. *: p<0.05.

### Blood leukocyte association with radiological progression of ILA and mortality

We explored association between peripheral blood leukocytes and their derived leukocyte indexes against radiographic progression and mortality using multivariate Cox proportional hazards models (table 3). All models included age, gender and the leukocyte values (or their derived indexes) contemporaneous with CT scan. Where stated, leukocyte coefficient of variation of each leukocyte type over the year leading to the CT was included in multivariate models. Nearest available blood measurement of monocytes, neutrophils and lymphocytes to first CT scan were obtained from standardised hospital “full blood count” measurements. Median (IQR) time interval between CT and nearest blood sampling was 1 (13–30) day.

**TABLE 3:** Multivariate Cox regression examining association between blood leukocytes and 1) mortality in early fibrotic interstitial lung abnormalities (EF-ILA) (n=1259) and 2) radiological progression in the EF-ILA cohort with available repeat computed tomography scans for comparison (n=362)

	**Mortality**		**Radiological progression**	
	**HR (95% CI)**	**p-value**	**HR (95% CI)**	**p-value**
**Age**	1.03 (1.01–1.06)	0.005*	1.03 (1.00–1.06)	0.027*
**Gender**	1.04 (0.76–1.42)	0.811	0.92 (0.67–1.27)	0.609
**Monocytes^#^**	1.12 (1.01–1.36)	0.003*	1.79 (1.05–2.86)	0.030*
**Neutrophils**	1.13 (1.07–1.19)	<0.001*	1.11 (1.02–1.29)	0.009*
**Lymphocytes**	0.97 (0.85–1.09)	0.574	0.99 (0.94–1.04)	0.596

HR: hazard ratio. ^#^: when adjusting for respiratory comorbidities, monocytes remained independently associated with progression (supplementary table S2). *: p<0.05.

In the 362 cases of EF-ILA that underwent at least two CT scans, monocyte count (HR 1.79, 95% CI 1.05–2.86; p=0.030) and neutrophil count (HR 1.11, 95% CI 1.02–1.29; p=0.009), MLR (HR 2.28, 95% CI 1.33–3.87; p=0.002), NLR (HR 1.07, 95% CI 1.01–1.14; p=0.024) and SIRI (HR 1.09, 95% CI 1.04–1.14; p=0.0002) were significantly associated with radiographic progression of the EF-ILA on multivariate Cox regression analysis (tables 3 and 4). Higher monocyte count, MLR, NLR and SIRI remained significant when adjusting for respiratory comorbidities (lung cancer, pneumonia and COPD/emphysema) in this cohort. Neutrophil count continued to show similar direction of effect towards progression, but was not significant (supplementary tables S2 and S3).

**TABLE 4:** Multivariate Cox regression examining association between blood leukocyte indexes and 1) mortality in early fibrotic interstitial lung abnormalities (EF-ILA) (n=1259) and 2) radiological progression in the EF-ILA cohort with available repeat computed tomography scans for comparison (n=362)

	**Mortality**		**Radiological progression**	
	**HR (95% CI)**	**p-value**	**HR (95% CI)**	**p-value**
**MLR**				
Age	1.03 (1.01–1.06)	0.006*	1.02 (0.99–1.05)	0.113
Gender	1.00 (0.74–1.36)	0.995	0.92 (0.67–1.27)	0.624
** **MLR	1.16 (1.02–1.31)	0.025*	2.28 (1.33–3.87)	0.002*
**NLR**				
Age	1.03 (1.01–1.06)	0.007*	1.02 (0.99–1.05)	0.122
Gender	0.98 (0.72–1.34)	0.910	0.96 (0.70–1.32)	0.814
NLR	1.07 (1.05–1.09)	<0.0001*	1.07 (1.01–1.14)	0.024*
**SIRI**				
Age	1.04 (1.01–1.06)	0.003*	1.03 (0.99–1.05)	0.079*
Gender	1.02 (0.75–1.38)	0.924	0.96 (0.69–1.31)	0.789
SIRI	1.06 (1.04–1.08)	<0.0001*	1.09 (1.04–1.14)	0.0002*

Each leukocyte index, age and gender is a separate model. Similar findings were observed when leukocyte indexes were adjusted for respiratory comorbidity and when adjusted for coefficient of variation over a year (supplementary tables S3 and S4). MLR: monocyte:lymphocyte ratio; NLR: neutrophil:lymphocyte ratio; SIRI: systemic inflammatory response ((monocytes×neutrophils) ÷ lymphocytes). *: p<0.05.

In the 1259 cases demonstrating EF-ILA on first CT, monocyte count (HR 1.12, 95% CI 1.01–1.36; p=0.003), neutrophil count (HR 1.13, 95% CI 1.07–1.19; p<0.001) and all their derived indexes were significantly associated with all-cause mortality: MLR (HR 1.16, 95% CI 1.02–1.31; p=0.025), NLR (HR 1.07, 95% CI 1.05–1.09; p<0.001) and SIRI (HR 1.06, 95% CI 1.04–1.08; p<0.001) (tables 3 and 4). Mortality risk was preserved in all models adjusting for respiratory comorbidity, except for monocytes (supplementary tables S2 and S3).

In separate regression models, coefficient of variation of longitudinal measurements for each leukocyte/index was also included, to adjust for any effect that variation in longitudinal measurement of these leukocyte parameters may have on clinical outcome. Monocytes maintained significant hazard towards both mortality and progression of EF-ILA (supplementary table S4). Distribution of leukocyte levels and their derived indexes are shown in supplementary figure S1.

## Discussion

This study shows that in an unselected cohort of patients undergoing thoracic CT scanning for all indications, in a 6-year period, 3.1% of patients showed evidence of EF-ILA. In a subset of patients who had more than one CT scan during this 6-year period, 43% progressed in extent of disease or demonstrated new traction bronchiectasis or honeycombing. Monocytes, MLR, NLR and SIRI were associated with progression in a multivariate analysis which included analysis of age and gender.

Our findings are comparable to ILA prevalence observed in large population-based cohorts [2, 3, 20] and lung cancer screening cohorts [21, 22] in which ILA ranged between 3% and 10%. Our definition of EF-ILA, which includes reticulation and GGO, but not traction bronchiectasis and honeycombing, probably encompassed “indeterminate UIP”, as defined in the 2018 IPF guidelines [17]. However, as we are unable to assess CT distribution of these ILAs in all cases for this large cohort, the terminology of EF-ILA was used. Since reticular abnormalities are common in older individuals and have previously been regarded as part of the normal spectrum of senescent lung [23], we limited inclusion to individuals aged 45–75 years at time of CT [24]. Comparable to other studies, gender was roughly of equal proportions across all cases in this cohort in those with EF-ILA, where traction bronchiectasis and honeycombing were excluded [2, 3].

The Age Gene/Environment Susceptibility (AGES) [3] and Framingham [2] population-based studies demonstrated ILA progression in 43% and 64% of cases, respectively, with associated risks of mortality similar to our cohort. In AGES, prevalence of indeterminate for UIP (iUIP) was estimated at 3.9%. iUIP was associated with mortality risk in univariate analysis (HR 1.6, p<0.0001), but nonsignificantly in multivariate analysis (HR 1.2, p=0.07). In our cohort, with a much higher number of cases, multivariate analysis showed that EF-ILA is associated with all-cause mortality (HR 1.87, p=0.002).

Importantly, we and others [6] demonstrate that radiographic progression is not observed in the majority of cases. It remains challenging to predict those cases that will progress to established fibrotic ILD. To address this, the Fleischner Society recently proposed a schema to facilitate triage, management and follow-up of ILAs [1]. This includes subcategorising cases according to ILA distribution on CT and presence (or absence) of ILAs indicative of established fibrosis.

ILA assessment in individuals with a history of familial ILD has identified associations with particulate exposures, age, positive smoking history, shorter telomere length and MUC5b risk allele [25, 26]. Associations among ageing-related biomarkers and ILA have been explored [27]; however, collectively these are costly and are not routinely available for large-scale use. Furthermore, with implementation of routine lung cancer screening pathways and greater use of thoracic CT for other diagnostic purposes, it is anticipated that ILA detection will increase. Patient follow-up could have a huge implication on clinical resources.

Peripheral blood leukocyte measurement is available as part of routine full blood count analysis and integrating this simple and cheap measure into risk stratification and ILA management is worthy of future consideration. This is supported by studies in IPF patients, where elevated peripheral blood monocyte count has been shown to be predictive of disease progression and mortality. The study by Scott *et al.* [13], a large multicentre retrospective cohort study, first demonstrated that monocyte counts ≥0.95×10^9^ cells·L^−1^ were associated with all-cause mortality in IPF and non-IPF fibrotic lung disease. Since then, other retrospective clinical studies have documented similar findings [14, 15, 28]. In an analysis of multiple independent cohorts (the Multi-Ethnic Study of Atherosclerosis (MESA), AGES, COPDGene and Evaluation of COPD Longitudinally to Identify Predictive Surrogate End-points (ECLIPSE); n=7396), Kim *et al.* [29] reported association between higher absolute monocyte count and ILA progression.

There are subtle, but important, differences between our study and that of Kim *et al.* [29], who reported the findings of a pooled analysis of four population-based cohorts (two COPD-focused cohorts). In this heterogeneous population, Kim *et al.* do not discuss whether their observed association between monocytes and ILA progression is limited to specific ILAs, such as those with early evidence of fibrosis. In our study, the inclusion criteria were biased towards selection of cases with CT features potentially compatible with early fibrosis, and were thus population-based enriched for patients potentially at risk of progressing to pulmonary fibrosis [1]. In our cohort of progressors, we detail the proportion of cases with new CT features representative of established pulmonary fibrosis and report association between absolute monocyte count (and other leukocyte parameters) with progression and all-cause mortality. In addition, we adopted a different approach to data analysis, employing Cox proportional hazards modelling to take account of time until events occurred.

Mechanistically, our findings could be explained by recent experimental evidence suggesting migration of monocytes from bone marrow to injured lung, then differentiating into macrophages with a pro-fibrotic phenotype [30]. Further support comes from translational studies that implicate distinct monocyte-derived alveolar macrophage populations in progression of fibrosis [31, 32].

Other studies have also implicated neutrophils and lymphocytes in pulmonary fibrosis. Akin to monocytes, neutrophils are recruited to areas of inflammation [33]. Similar to macrophages, they can alter their microenvironment by secreting proteases, oxidants, cytokines and chemokines [34]. Additionally, neutrophils are a substantial source of matrix metalloproteinases which are involved in collagen deposition and extracellular matrix formation [35]. Neutrophilic bronchoalveolar lavage specimens taken from patients with IPF have been associated with early mortality [36].

We have previously demonstrated in a cohort of patients with CT scans demonstrating iUIP that peripheral blood monocyte and neutrophil counts are implicated in progression to IPF [37], and the association of NLR, MLR and SIRI with mortality in IPF [38]. MLR has been used primarily to prognosticate in cancer studies in recognition that host systemic inflammatory responses influence tumour proliferation and disease progression [39]. NLR has been heavily studied as a systemic inflammatory marker [40]. It has been used to prognosticate systemic inflammatory diseases such as rheumatoid arthritis [41], and recently in connective tissue disease-related ILD and IPF [16]. SIRI integrates neutrophils, monocytes and lymphocytes into one composite measure and has shown promise as a prognosticator within oncology [19].

There are several limitations that should be considered when interpreting these results. We were unable to quantify extent of disease and extent of progression. Categorisation was based on qualitative information extracted from radiology reports and does not quantify individual ILA extent, which may contribute to rate of progression. Similarly, we were unable to capture indications for CT and we cannot account for clinical symptoms, which would have been interesting to explore. Therefore, interpretation of our findings is limited to association between CT features with blood leukocytes. The patients were selected because of their need for a thoracic CT, so the true prevalence in the population is unknown, only in those who requires a thoracic CT. In the group where we assessed progression, the interval between the first and last CT was longer in those who progressed compared to those who did not. It could be argued that those who did not progress would do so over a longer period of assessment. The nil-ILA group was identified by description of negative findings of keywords from CT reports (*e.g.* “no reticulation”). We acknowledge that this represents a smaller proportion of patients with normal CT scans and may have introduced bias to EF-ILA mortality risk calculation. However, selection criteria were identical and demographic and comorbidity profiles were comparable between nil-ILA and EF-ILA group. Furthermore, demographic profiles of the nil-ILA group are also comparable to the nil-ILA cohorts of other longitudinal studies [3].

A proportion of our EF-ILA cohort were subsequently seen in our ILD clinic. Mean time from first CT scan demonstrating EF-ILA to ILD clinic attendance was +3.1 years. As the ILD clinic was a first-attender clinic, it is likely (but not verified) that these were patients who became symptomatic or demonstrated progression after a follow-on CT scan and did not have a prior diagnosis of ILD. Therefore, there is a possibility of prior undiagnosed ILD. Under-reporting of ILA has been described previously [42]; as such, we cannot exclude the possibility that a degree of misclassification may have occurred. However, 80.7% of CT scans were reported by post-radiology fellowship specialist thoracic radiologists who attend ILD multidisciplinary team meetings, based at a single centre, which might mitigate interobserver difference. Excellent interobserver correlation between our thoracic radiologists in reporting ILD features (r=0.91; p<0.001) has been described previously [43]. The remaining 19.3% of CTs were reported by one out of 14 Oxford-based post-training radiologists. All radiologists collectively agree on descriptive reporting phrases, and there are regular local discrepancy meetings to check on accuracy of reporting.

World Health Organization International Classification of Diseases version 10 (ICD-10) coding was used to identify comorbidity during the 6 years of study follow-up, but without coding dates we were unable to align comorbidity events to first CT scan date. Smoking history was poorly captured using “ICD-10 coding”. Therefore, we elected not to include this information in our multivariate analysis, but acknowledge that patients who smoke may show a greater rate of ILA progression. Finally, the single-centre and retrospective nature of this study should be taken forward by prospective, intervention and validation studies in a different cohort.

Notwithstanding these limitations, our study, in a very large cohort with high proportion of specialist thoracic radiologist reporting, demonstrates that monocyte levels, MLR, NLR and SIRI are associated with progression in EF-ILA. Further prospective studies will help determine if these parameters could be used to help prioritise patients who might benefit from follow-up.

## Supplementary material

10.1183/23120541.00226-2022.Supp1 **Please note:** supplementary material is not edited by the Editorial Office, and is uploaded as it has been supplied by the author.
Supplementary material 00226-2022.SUPPLEMENT
