# Supplementary material for: Increased monocyte level is a risk factor for radiological progression in patients with early fibrotic interstitial lung abnormality
Source: ERJ Open Res. 2022 Jul 4;8(3):00226-2022. doi: 10.1183/23120541.00226-2022 (PMC9251369; doi:10.1183/23120541.00226-2022)
Supplement: Supplementary file 1 [file 00226-2022.SUPPLEMENT.pdf]

Supplementary materials

Description of methods

Search Criteria and data collation

The Clinical Record Interactive Search (CRIS) database of the Oxford University Hospitals NHS trust was used to search for reports for all thorax-protocolised CT scans performed between January-2015 and December-2020. The search was conducted in two phases. In phase 1, search criteria were selective for the early fibrotic ILA radiological pattern which we defined as reticulation in absence of traction bronchiectasis and honeycomb formation. Search criteria is as follows:

["reticulation" or "interstitial"]
AND
["sub-pleural" or "basal" or "lower zone" or "Possible UIP"]
AND
[Age range: 45-75]

Search criteria were defined with close guidance from our lead thoracic radiologist (RB) based upon the vocabulary tendencies of our thoracic radiologists when reporting CTs. We defined upper limit of age as 75 years.[1] Reticular abnormalities are common in older individuals and have sometimes been regarded as part of the normal spectrum of senescent lung.[2] Reporting findings as such could downgrade its clinical significance and introduce ambiguity to this dataset.[3] Possible UIP was included in search criteria as this term, used in the 2011 iteration of the IPF guideline, can also include reticulation in absence of traction bronchiectasis and distinct absence of honeycombing.[4] Fibrotic ILAs with basal and peripheral predominance are considered to possess greater risk of progression and mortality. As such this distribution was also incorporated into the search criteria in an "OR" rather than "AND" fashion. This was to maximise the initial search results and also because it has previously been documented that under-reporting of ILA has previously been described.[5]

In Phase 2, qualitative information was extracted from CT reports (identified from above search criteria) and converted into structured binary data. Additional parenchymal features were screened for in CT scan reports, adjudicated and collated. Features included ground glass opacities (GGO), emphysema, traction bronchiectasis and honeycombing.[6,7] To ensure we . Specific combinations of concurrent parenchymal features (e.g. reticulation and GGO) were also collated. Importantly, traction bronchiectasis and honeycombing are considered representative of established fibrotic ILD and therefore cases with co-existing traction bronchiectasis and honeycombing identified in phase 2 were partitioned from cases of EF-ILA. Where feasible cases with reticulation and or traction bronchiectasis and or honeycombing were sub-classified into UIP categories to communicate ILA extent and co-existence to the readership.[8] This does not assume cases categorised in this manner have IPF. Similarly, cases with reticulation were subcategorised into cases with and without other non-fibrotic parenchymal abnormalities including GGO. Cases identified from the preliminary key word search (phase 1), however later found on screening (phase 2) that CT reports were detailing negative / absence of specific radiological patterns were defined as a ' Nil ILA' reference cohort.

Radiographic progression was recorded as a binary event based on presence of new or increase in pre-existing parenchymal features. It was captured based upon text-based searching of CT report descriptions. Progression was adjudicated based upon perusal of all CT reports of repeat CTs by one specialist trainee (AA) using pre-defined criteria. Progression was defined as increase in either extent of identified early fibrotic features (reticulation and or GGO), new emergence of traction bronchiectasis, and/or new emergence of honeycombing on follow on CT. In cases not

demonstrating radiographic progression, this was defined as unchanged pre-existing parenchymal features and absence of new features.

Total number of subjects that underwent a thorax-protocolised CT between January-2015 and December-2020 was used as a denominator to estimate EF-ILA prevalence in cases indicated to undergo thoracic CT.

CT reporting

CT scans were assessed using text-based searching of CT reports. 80.9% of CTs were reported by 1 of 8 thoracic radiologists. All were consultants (with UK Certificate of Completion of Training/CCT for Radiology) and 7 of 8 of these consultants also held a post -CCT Thoracic Radiology Fellowship and participated in dedicated Interstitial Lung Disease MDTs. The other thoracic radiologist has >20 years of experience in reporting HRCT for ILD in our centre. The remainder were reported by one of 14 Oxford-based post CCT radiologists. Our thoracic radiologists have contributed to >20 radiology-based studies over the last decade. In one study, performed with our research group, the agreement between of reporting ILD features between the two radiologists was excellent ($r=0.91$; $p<0.001$ by Pearson's Correlation, also tested by Bland Altman; Suppl Fig 4A).[9]

Comorbidity profiles

Comorbidities for each case were search for using ICD-10 coding. Specific ICD-10 codes,[10] pertaining to respiratory and non-respiratory diagnoses were cross-referenced with electronic health records. Similarly, attendance at ILD clinic, date thereof and date of mortality obtained and time intervals between these events and CT date deduced. Comorbidities are listed as binary events. It was not possible to obtain date of comorbidity diagnoses for all cases and therefore not possible to determine which diagnoses were established prior to, at or after first CT scan showing ILA. For this reason, comorbidities were not included in all multivariate analysis and interpretation of this data is limited to association rather than causality.

Statistical analysis

Cox proportional hazard models were used to determine hazard ratios (HRs) to progression and all-cause mortality (separate models). In both models, age, gender, monocytes, neutrophils, and lymphocytes levels obtained at a time point closest to the CT scan were included. CoV values for each case were calculated from available counts for monocyte, neutrophil, and lymphocytes (and derived indexes) in the 1 year up to first CT to account for within-group variance in these measures. In separate models, CoV of longitudinal measurements for each leukocyte / index (for each subject) was included in regression models, along with the leukocyte value closest to CT. This was to adjust for any effect that variation in longitudinal measurement of these leukocyte parameters may have on clinical outcome, relative to the corresponding leukocyte value closest to CT used in the model.

Table S1

Covariate	n (%)	Death (%)	Mortality HR (95% CI)	Sig.
Age	--	--	1.01 (0.99-1.03)	0.107
Gender (Male)	1486 (54.3%)	268 (18.0%)	1.09 (0.88-1.40)	0.425
Lung cancer	269 (9.8%)	154 (57.2%)	4.98 (3.96.23)	<0.001*
Pneumonia	657 (24.0%)	251 (38.2%)	3.05 (2.45-3.81)	<0.001*
COPD / Emphysema	569 (20.8%)	165 (28.9%)	1.14 (0.90-1.43)	0.278
Nil ILA (reference)	355 (12.9%)	43 (12.1%)	--	--
Early fibrotic ILA	1259 (46.0%)	183 (14.5%)	1.52 (1.01-2.27)	0.043*
TBx without Honeycombing	490 (17.9%)	86 (17.6%)	1.70 (1.11-2.62)	0.016*
Honeycombing +/- TBx	272 (9.9%)	87 (32.0%)	3.12 (2.03-4.81)	<0.001*

Table S1 Multivariate Cox regression examining association of ILA features on first CT scan with mortality. Hazard ratios (HR) for ILA categories representative of risk relative to Nil ILA reference category. TBx; Traction bronchiectasis, EF-ILA; Early-fibrotic ILA. Model adjusted for age, gender and respiratory co-morbidity (lung cancer, pneumonia and COPD/emphysema).

Table S2

	Mortality		Radiological progression	
Covariates	HR (95% CI)	Sig.	HR (95% CI)	Sig.
-Age	1.02 (1.01-1.03)	0.015*	1.03 (1.00-1.06)	0.038*
-Gender	1.00 (0.82-1.22)	0.977	0.91 (0.66-1.26)	0.573
-Pneumonia	2.97 (2.40-3.67)	<0.001*	1.38 (0.99-1.32)	0.060
-COPD / Emphysema	1.12 (0.90-1.39)	0.294	0.64 (0.44-0.94)	0.023*
-Lung cancer	4.46 (3.61-5.51)	<0.001*	1.27 (0.80-2.00)	0.309
-Monocytes ($1x10^9$/l)	1.15 (0.85-1.55)	0.376	1.72 (1.10-2.69)	0.018*
-Neutrophils ($1x10^9$/l)	1.07 (1.04-1.10)	<0.001*	1.05 (0.98-1.12)	0.154
-Lymphocytes ($1x10^9$/l)	0.79 (0.70-0.90)	<0.001*	0.99 (0.95-1.03)	0.690

Table S2 Multivariate cox regression examining association between blood leukocyte and (i) mortality in "early fibrotic" ILA (n=1259) and (ii) radiological progression in "early fibrotic" ILA cohort with available repeat CT for comparison (n=362). Leukocytes adjusted for age, gender and respiratory comorbidities of COPD, lung cancer and pneumonia.

Table S3

Covariates	Mortality HR (95% CI)	Mortality Sig.	Radiological progression HR (95% CI)	Radiological progression Sig.
**MLR**				
-Age	1.02 (0.99-1.05)	0.059	1.02 (0.99-1.05)	0.161
-Gender	0.97 (0.71-1.33)	0.858	0.90 (0.66-1.25)	0.550
-Pneumonia	2.80 (2.02-3.87)	<0.001*	1.27 (0.91-1.79)	0.166
-COPD / Emphysema	1.31 (0.94-1.82)	0.114	0.66 (0.45-0.98)	0.038*
-Lung cancer	5.87 (4.29-8.02)	<0.001*	1.33 (0.84-2.11)	0.219
-MLR	1.21 (1.04-1.39)	0.011*	2.07 (1.19-3.62)	0.010*
**NLR**				
-Age	1.02 (1.01-1.05)	0.044*	1.02 (0.99-1.05)	0.160
-Gender	0.94 (0.69-1.28)	0.698	0.94 (0.68-1.29)	0.687
-Pneumonia	2.58 (1.97-3.58)	<0.001*	1.27 (0.90-1.80)	0.177
-COPD / Emphysema	1.31 (0.94-1.82)	0.113	0.69 (0.47-1.01)	0.054
-Lung cancer	6.17 (4.51-8.44)	<0.001*	1.40 (0.88-2.22)	0.153
-NLR	1.07 (1.05-1.09)	<0.001*	1.07 (1.01-1.14)	0.019*
**SIRI**				
-Age	1.03 (1.01-1.05)	0.034*	1.02 (1.00-1.05)	0.109
-Gender	0.98 (0.72-1.33)	0.883	0.94 (0.68-1.28)	0.676
-Pneumonia	2.75 (1.99-3.81)	<0.001*	1.33 (0.95-1.85)	0.096
-COPD / Emphysema	1.30 (0.93-1.81)	0.124	0.65 (0.44-0.96)	0.030*
-Lung cancer	5.69 (4.16-7.79)	<0.001*	1.32 (0.83-2.08)	0.241
-SIRI	1.03 (1.01-1.06)	0.007*	1.09 (1.04-1.14)	<0.001*

Table S3 Multivariate cox regression examining association between blood leukocyte indexes and (i) mortality in "early fibrotic" ILA (n=1259) and (ii) radiological progression in "early fibrotic" ILA cohort with available repeat CT for comparison (n=362). Leukocytes adjusted for age, gender and respiratory comorbidities of COPD, lung cancer and pneumonia.

Table S4

Covariates	Mortality HR (95% CI)	Mortality Sig.	Radiological progression HR (95% CI)	Radiological progression Sig.
**Full blood count**				
-Age	1.03 (1.01-1.06)	0.007*	1.03 (1.01-1.06)	0.023*
-Gender	0.96 (0.71-1.31)	0.797	0.937 (0.68-1.30)	0.700
-Monocytes (1x10^9/l)	1.18 (1.04-1.33)	0.011	1.74 (1.15-2.64)	0.009*
-Monocyte CoV	7.37 (3.85-14.3)	<0.0001*	7.43 (2.84-19.44)	<0.0001*
-Lymphocytes (1x10^9/l)	0.95 (0.84-1.07)	0.395	0.99 (0.95-1.03)	0.652
-Lymphocytes CoV	8.88 (4.72-16.4)	<0.0001*	7.50 (2.77-20.31)	<0.0001*
-Neutrophils (1x10^9/l)	1.08 (1.04-1.13)	<0.0001*	1.05 (0.99-1.12)	0.127
-Neutrophils CoV	4.42 (2.68-7.31)	<0.0001*	2.75 (1.28-5.90)	0.009*
**MLR**				
-Age	1.04 (1.01-1.06)	0.003*	1.03 (0.99-1.06)	0.065
-Gender	0.983 (0.72-1.34)	0.915	0.90 (0.65-1.24)	0.535
-MLR	1.05 (0.93-1.20)	0.441	2.03 (1.17-3.56)	0.013*
-MLR CoV	3.25 (2.42-4.37)	<0.0001*	3.08 (1.43-6.68)	0.004*
**NLR**				
-Age	1.04 (1.01-1.06)	0.002*	1.03 (0.99-1.06)	0.075
-Gender	0.97 (0.72-1.32)	0.859	0.95 (0.70-1.31)	0.100
-NLR	1.05 (1.03-1.07)	<0.0001*	1.08 (1.01-1.56)	0.026*
-NLR CoV	2.59 (1.83-3.59)	<0.0001*	1.41 (0.79-2.77)	0.317
**SIRI**				
-Age	1.04 (102-1.07)	0.001*	1.03 (0.99-1.06)	0.058
-Gender	1.04 (0.76-1.41)	0.826	0.95 (0.69-1.30)	0.731
-SIRI	1.04 (1.02-1.07)	<0.0001*	1.08 (1.03-1.14)	0.001*
-SIRI CoV	2.54 (1.93-3.37)	<0.0001*	1.62 (1.04-2.54)	0.032*

Table S4 Multivariate cox regression examining association between blood leukocyte indexes and (i) mortality in "early fibrotic" ILA (n=1259) and (ii) radiological progression in "early fibrotic" ILA cohort with available repeat CT for comparison (n=362). Covariates in "full blood count", MLR, NLR and SIRI models adjusted for age, gender, and leukocyte co-efficient of variation (CoV).

Figure S1

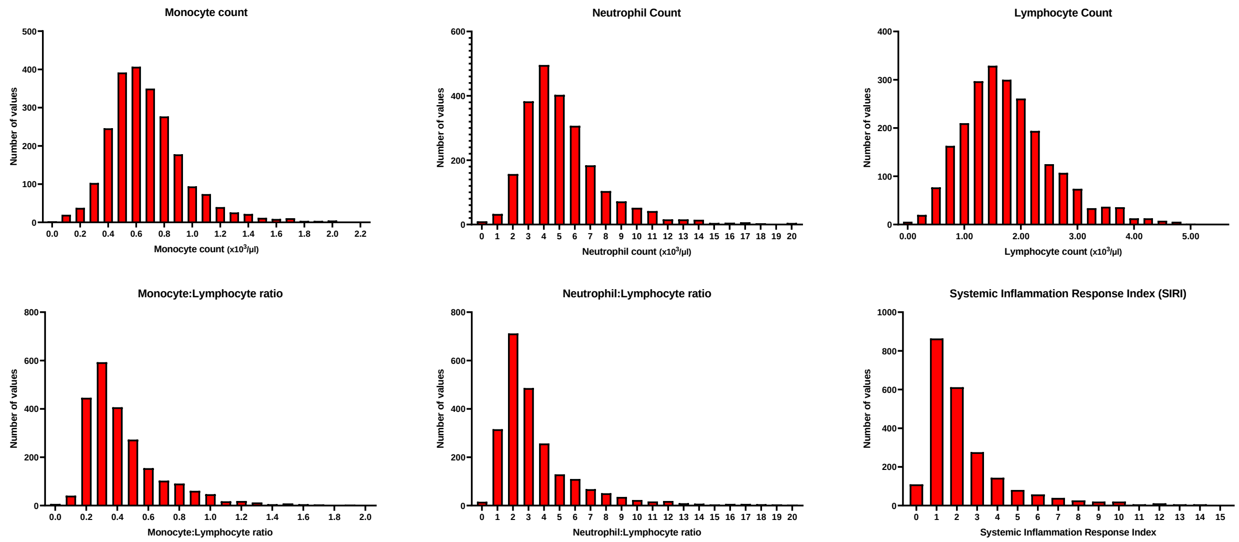

Figure S1. Histograms demonstrating Leukocyte distribution (closest value to CT)

References

1. Copley SJ, Wells AU, Hawtin KE, et al. Lung morphology in the elderly: comparative CT study of subjects over 75 years old versus those under 55 years old. *Radiology.* 2009;251(2):566-573.
2. Copley SJ. Morphology of the Aging Lung on Computed Tomography. *J Thorac Imaging.* 2016;31(3):140-150.
3. Meiners S, Eickelberg O, Königshoff M. Hallmarks of the ageing lung. *European Respiratory Journal.* 2015;45(3):807-827.
4. Raghu G, Collard HR, Egan JJ, et al. An official ATS/ERS/JRS/ALAT statement: idiopathic pulmonary fibrosis: evidence-based guidelines for diagnosis and management. *Am J Respir Crit Care Med.* 2011;183(6):788-824.
5. Oldham JM, Adegunsoye A, Khera S, et al. Underreporting of Interstitial Lung Abnormalities on Lung Cancer Screening Computed Tomography. *Ann Am Thorac Soc.* 2018;15(6):764-766.
6. Hatabu H, Hunninghake GM, Richeldi L, et al. Interstitial lung abnormalities detected incidentally on CT: a Position Paper from the Fleischner Society. *The Lancet Respiratory Medicine.* 2020;8(7):726-737.
7. Hansell DM, Bankier AA, MacMahon H, McLoud TC, Müller NL, Remy J. Fleischner Society: glossary of terms for thoracic imaging. *Radiology.* 2008;246(3):697-722.
8. Raghu G, Remy-Jardin M, Myers JL, et al. Diagnosis of Idiopathic Pulmonary Fibrosis. An Official ATS/ERS/JRS/ALAT Clinical Practice Guideline. *Am J Respir Crit Care Med.* 2018;198(5):e44-e68.
9. Benamore R, Kendrick YR, Repapi E, et al. CTAS: a CT score to quantify disease activity in pulmonary sarcoidosis. *Thorax.* 2016;71(12):1161-1163.
10. World Health O. ICD-10 : international statistical classification of diseases and related health problems : tenth revision. In. 2nd ed ed. Geneva: World Health Organization; 2004.
